# Polymyxin Resistance in *Acinetobacter baumannii*: Genetic Mutations and Transcriptomic Changes in Response to Clinically Relevant Dosage Regimens

**DOI:** 10.1038/srep26233

**Published:** 2016-05-19

**Authors:** Soon-Ee Cheah, Matthew D. Johnson, Yan Zhu, Brian T. Tsuji, Alan Forrest, Jurgen B. Bulitta, John D. Boyce, Roger L. Nation, Jian Li

**Affiliations:** 1 Drug Delivery, Disposition and Dynamics, Monash Institute of Pharmaceutical Sciences, Monash University (Parkville campus), 381 Royal Parade, Parkville, Victoria 3052, Australia; 2 Laboratory for Antimicrobial Pharmacodynamics, Department of Pharmacy Practice, University of Buffalo, Kapoor Hall, Buffalo, NY 14214-8033, USA; 3 Division of Pharmacotherapy and Experimental Therapeutics, University of North Carolina Eshelman School of Pharmacy, Genetic Medicine Building, 120 Mason Farm Road, Chapel Hill NC 27599, USA; 4 Center for Pharmacometrics and Systems Pharmacology, Department of Pharmaceutics, College of Pharmacy, University of Florida, 6550 Sanger Road, Orloando FL 32827, USA; 5 Biomedicine Discovery Institute and Department of Microbiology, School of Biomedical Sciences, Monash University (Clayton campus), Wellington Road, Clayton, Victoria 3800, Australia

## Abstract

Polymyxins are often last-line therapeutic agents used to treat infections caused by multidrug-resistant *A. baumannii*. Recent reports of polymyxin-resistant *A. baumannii* highlight the urgent need for research into mechanisms of polymyxin resistance. This study employed genomic and transcriptomic analyses to investigate the mechanisms of polymyxin resistance in *A. baumannii* AB307-0294 using an *in vitro* dynamic model to mimic four different clinically relevant dosage regimens of polymyxin B and colistin over 96 h. Polymyxin B dosage regimens that achieved peak concentrations above 1 mg/L within 1 h caused significant bacterial killing (~5 log_10_CFU/mL), while the gradual accumulation of colistin resulted in no bacterial killing. Polymyxin resistance was observed across all dosage regimens; partial reversion to susceptibility was observed in 6 of 8 bacterial samples during drug-free passaging. Stable polymyxin-resistant samples contained a mutation in *pmrB*. The transcriptomes of stable and non-stable polymyxin-resistant samples were not substantially different and featured altered expression of genes associated with outer membrane structure and biogenesis. These findings were further supported *via* integrated analysis of previously published transcriptomics data from strain ATCC19606. Our results provide a foundation for understanding the mechanisms of polymyxin resistance following exposure to polymyxins and the need to explore effective combination therapies.

The antimicrobial resistance crisis has become a significant threat to public health^1^. Globally, hospital outbreaks of infections caused by multi-drug resistant (MDR) Gram-negative pathogens, such as *Acinetobacter baumannii*, are being increasingly reported^2^,^3^. With few novel antibiotics in late-stage clinical development, clinicians may soon be left with no options for the treatment of recalcitrant infections caused by these MDR pathogens. *A. baumannii* has emerged as a particularly problematic pathogen, owing to its propensity to acquire resistance to most currently available antibiotics^4^. Polymyxins (*i.e.* polymyxin B and colistin) are used as a salvage therapy for *A. baumannii* infections where susceptibility testing suggests that carbapenems and aminoglycosides are unlikely to be effective^5^,^6^,^7^. Owing to their clinical introduction in the 1950s and fall from favour a decade or so later, the pharmacology of polymyxins has not been as thoroughly investigated as for modern antibiotics, until recently. While polymyxins demonstrate *in vitro* activity against many MDR *A. baumannii* bacterial isolates^3^,^8^,^9^, reports of polymyxin-resistant *A. baumannii* clinical isolates^10^ highlight an urgent need to investigate the influence of polymyxin dosage regimens on the emergence of resistance.

Polymyxins are cationic amphipathic compounds, containing a cyclic heptapeptide ring joined to a fatty acyl tail by a linear tripeptide. The L-2,4-diaminobutyric acid residues give rise to the cationic and hydrophilic nature of polymyxins, while the fatty acyl tail and position 6/7 amino acids of the heptapeptide ring contribute to the hydrophobicity of the compounds^11^. The aforementioned physicochemical properties of polymyxins are critical for their initial interaction with the negatively charged moieties and hydrophobic regions of lipid A of lipopolysaccharide (LPS) within the bacterial outer membrane (OM), leading to its permeabilisation^11^. While the interaction between lipid A and polymyxins is well characterised and essential for their ultimate bactericidal effect^11^, the mechanism of polymyxin killing following perturbation of the OM has yet to be fully elucidated^12^,^13^,^14^,^15^,^16^. To date, two mechanisms of polymyxin resistance have been identified in *A. baumannii*: modification of lipid A with phosphoethanolamine and/or galactosamine and the complete loss of LPS from the OM^17^,^18^,^19^,^20^,^21^. Current literature suggests that both mechanisms of resistance abolish polymyxin-induced bacterial killing by preventing the interaction of polymyxins with the OM, and are mediated by the *pmrCAB* operon^17^,^21^ (for lipid A modification with phosphoethanolamine), *naxD*^20^ (for modification with galactosamine) or *lpx* biosynthetic cluster (for LPS loss)^19^. There is a paucity of knowledge on the emergence and mechanism(s) of resistance in response to the polymyxin exposure profiles associated with clinically relevant dosage regimens of colistin and polymyxin B.

The two clinically used polymyxins, colistin and polymyxin B, differ in their administered forms and exhibit markedly different clinical pharmacokinetics (PK)^5^. Colistin is administered parenterally as the sodium salt of its inactive pro-drug colistin methanesulphonate (CMS), while polymyxin B is available in the clinic as the sulphate salt of its active form. Following administration, CMS is converted slowly to colistin while simultaneously undergoing rapid renal elimination, which leads to a delay in the attainment of target colistin concentrations^22^,^23^,^24^. In contrast, the administration of polymyxin B enables target concentrations to be more rapidly achieved^25^. Although colistin and polymyxin B are considered equivalent based upon their antimicrobial activity *in vitro*^26^, it was hypothesised that differences in their plasma concentration *versus* time profiles following initiation of therapy with CMS and polymyxin B, respectively, are likely to substantially affect their pharmacodynamic responses in patients. The objectives of this study were to investigate the transcriptomic profile and stability of polymyxin resistance in *A. baumannii* when exposed in an *in vitro* dynamic model to clinically relevant concentration *versus* time profiles of colistin and polymyxin B.

## Methods

### Bacterial strain and media

*A. baumannii* strain AB307-0294, a previously characterised polymyxin-susceptible (MIC: 1.0 mg/L) clinical isolate belonging to international clonal complex I^27^,^28^, was investigated in this study. Cation-adjusted Mueller-Hinton broth (CAMHB, Oxoid, Ca^2+^: 20–25 mg/L, Mg^2+^: 10–15 mg/L) was used in both the *in vitro* dynamic model and subsequent passaging. All bacterial cultures, including starter cultures and the *in vitro* model, were maintained at 37 °C for the duration of the experiment.

### *In vitro* model and passaging in drug-free broth

**Figure 1:**
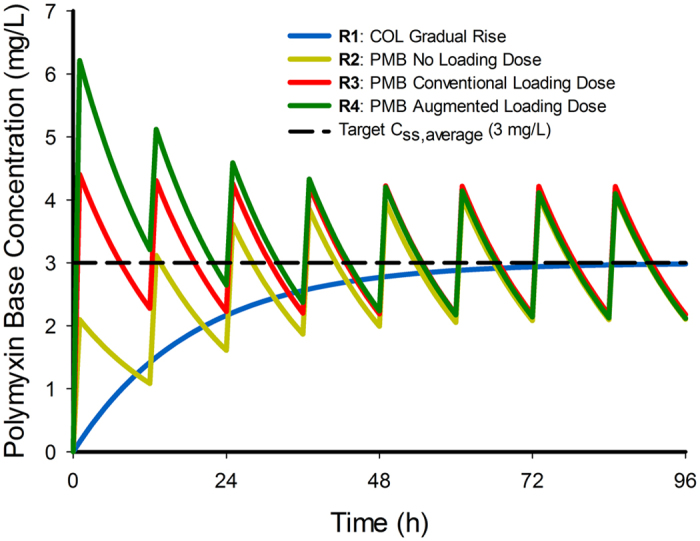


A starting inoculum of 10^6^ CFU/mL of log-phase bacteria cultured from a single colony was introduced into a previously described *in vitro* one-compartment model (IVM). This model allows clinically relevant concentration *versus* time profiles of an antibiotic to be accurately achieved in a central reservoir inoculated with the organism of interest^29^. A total of 4 concentration-time profiles were simulated in the IVM (Fig. 1) with a central reservoir volume of 250 mL. These profiles corresponded to: the gradual accumulation of colistin as would be seen at the initiation of CMS therapy with no loading dose^22^ (regimen 1); 1-h polymyxin B infusion every 12 h without a loading dose (regimen 2); as for regimen 2 but with a loading dose to achieve the steady state immediately (regimen 3); and, regimen 2 initiated with an augmented loading dose to achieve concentrations over the first several hours higher than the eventual steady-state concentrations (regimen 4). Each regimen and the growth control were conducted in two replicates. For all regimens, an elimination half-life of 11.6 h was applied for both colistin and polymyxin B, representative of pharmacokinetic behaviour of both polymyxins in critically-ill patients^22^,^23^,^25^,^30^. For all four regimens an average steady-state concentration of 3 mg/L was simulated; for regimen 4, the augmented loading dose achieved a peak polymyxin B concentration of 6 mg/L after this initial dose and subsequently the concentrations declined to achieve the same steady-state profile as for the other two polymyxin B regimens (Fig. 1). The elimination half-life and target polymyxin concentrations were selected to mimic the disposition of polymyxin B and colistin in critically-ill patients^22^,^25^,^23^. Samples (1 mL) were collected from the central reservoir at 0, 1, 8, 23, 28, 47, 52, 71 and 96 h, and numbers of viable bacteria were determined by plating onto drug-free agar plates. Population analysis profiles (PAPs) were obtained at 23, 47, 71 and 96 h by counting viable bacteria after plating cultures on polymyxin B containing agar plates (2, 4 and 8 mg/L as sulphate). At the conclusion of the IVM (96 h), polymyxin-resistant bacterial cells were isolated from each of the treated reservoirs (n = 8 total) and passaged daily for a further 96 h in drug-free CAMHB. PAPs were obtained daily on polymyxin B-containing (1, 2, 4 and 8 mg/L) agar plates.

### Genomics and transcriptomics

Genomics samples were collected at the conclusion of passaging in drug-free CAMHB (192 h; consisting of 96 h in IVM and 96 h drug-free passaging) and DNA was prepared using a QIAamp DNA mini kit (Qiagen, USA) for high-throughput sequencing (150 bp paired-end reads). Transcriptomic profiling was performed on cultures recovered from each reservoir (n = 10; including growth control) at the conclusion of the IVM (96 h). Cultures collected for transcriptomic profiling (containing ~10^9^ CFU per sample) were centrifuged at 9000 × *g* (4 °C) for 10 min and resuspended in 1 mL of RNALater (Qiagen, USA) for 10 min before a second centrifugation at 5000 × *g* for 10 min, with the pellet stored at −80 °C prior to sequencing. Total RNA was purified from each sample (Qiagen RNeasy; Qiagen, USA), ribosomal RNAs removed (Ribo-Zero rRNA removal kit; Illumina, UK) and libraries prepared for RNA sequencing (100 bp single-end reads) as previously described^31^. DNA and RNA sequencing was performed on an Illumina HiSeq (Medical Genomics Facility, Monash Health Translation Precinct, Monash University, Victoria, Australia). RNA sequencing was performed over two Illumina HiSeq lanes, with replicates for each treatment condition analysed on separate lanes.

### Next-generation sequencing data analysis

Illumina HiSeq reads for both genomic and transcriptomic analyses were clipped using the Nesoni software package (Victorian Bioinformatics Consortium) before mapping to a previously published^27^ genome for *A. baumannii* AB307-0294 (Genbank accession: NC_011595) using the Short Read Mapping Package (SHRiMP 2.2.3). The average number of reads per sample was ~5 million for the genomic analysis, and ~23 million mapped reads per sample for transcriptomics analysis. Single nucleotide polymorphisms (SNPs) in the genomic and transcriptomic data were identified with Freebayes^32^, using the previously published genome for *A. baumannii* AB307-0294 as a reference. Differential gene expression analysis of transcriptomic data was performed in Degust (www.vicbioinformatics.com/degust), a visual interface for the Voom and Limma R packages^33^. Statistical significance of differential gene expression was calculated using the F-statistic, jointly considering all treatment groups and adjusted using the Benjamini Hochberg method to control the false discovery rate (FDR)^34^. Differential expression was defined as a log_2_ fold-change (log_2_FC) of >1.0 in any of the treatment groups relative to the growth control with a corresponding FDR of <0.05. Interproscan^35^ (version 5) was used for functional and gene ontology (GO) term annotation of the published *A. baumannii* AB307-0294 genome^27^. Principal component and GO term enrichment analyses (Fisher’s exact test) were performed in R.

### Integrated gene expression analysis for comparison of data from closely related strains

In addition to the conventional differential gene expression analysis, a sparse partial least squares regression discriminant analysis^36^ (SPLS-DA) model was constructed to identify gene expression patterns that were (1) unique to the early stages of polymyxin exposure (≤1 h), and (2) shared between early- and late-stage polymyxin exposure (1 and 96 h). Matching of orthologous genes, defined as gene pairs with a p-value of <10^−5^ when clustered using OrthoMCL^37^, was used to merge data from the present study with a previously published transcriptomic dataset from *A. baumannii* strain ATCC 19606 sampled 15 and 60 min following colistin, doripenem, or colistin/doripenem combination treatment^31^. In total, 16 samples consisting of 5 untreated controls, 6 polymyxin-treated, 3 doripenem-treated and 2 combination-treated samples were used from the previously published dataset^31^. A variance stabilisation transformation was performed on the combined data in R using the DESeq2^38^ package prior to SPLS-DA.

### SPLS-DA model validation

The SPLS-DA model was subjected to k-fold (k = 2) validation to select the smallest number of genes that optimally described the biological variation within the combined dataset. The combined data from the present study and our previous paper^31^ were randomly partitioned into two segments (n = 13 each), with each segment used individually for model construction and the combined dataset used to determine the classification error rate (two tests per partitioning). The error rate for each candidate model was calculated as the average of 300 trials (600 tests) to account for the stochastic nature of partitioning. Candidate models contained between 10 and 150 genes (10 gene increments; 300 error rate trials per model) and were evaluated by their corresponding error rates. Once the smallest number of genes for inclusion had been determined, a second k-fold (k = 6; 6 tests per partitioning) validation was performed (50 trials; 300 tests total) to identify the inclusion rate of individual genes within models constructed on partitioned data sets.

## Results

### Characterisation of polymyxin activity and resistance in *A. baumannii*

**Figure 2:**
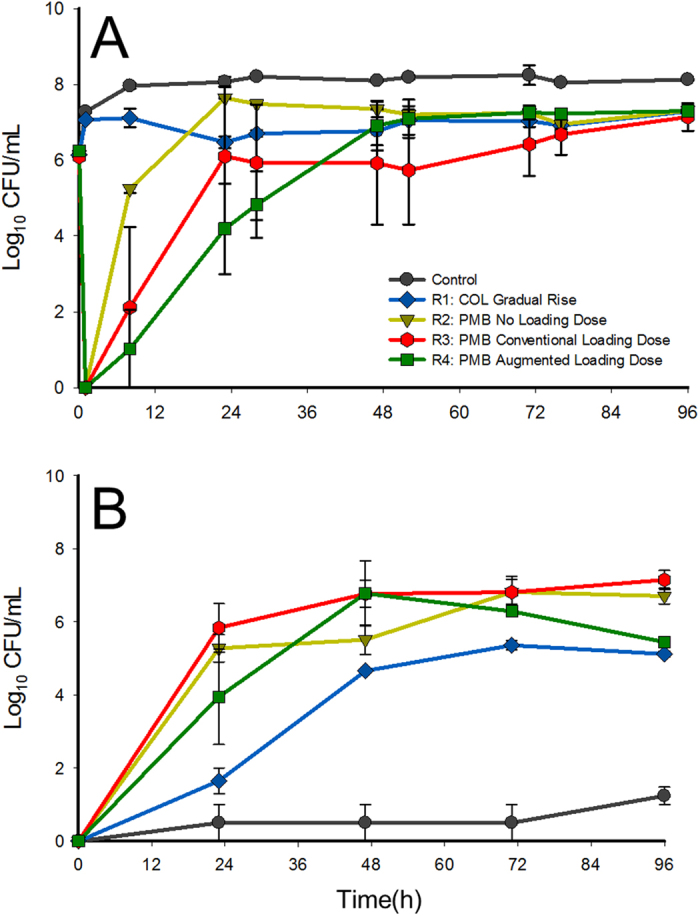
Viable counting results (mean ± SD; *n* = 2) for *A. baumannii* AB307-0294 samples grown on (**A**) drug-free agar plates and (**B**) polymyxin B containing (8 mg/L) agar plates.

Polymyxin B dosage regimens that rapidly attained concentrations >1 mg/L exhibited more bacterial killing compared to the simulated colistin dosing regimen (Fig. 2A). The development of polymyxin resistance was phenotypically similar across all dosage regimens (Fig. 2B). At the conclusion of polymyxin B or colistin treatment (96 h), bacterial cells isolated from the treated IVM reservoirs showed only a ~1–2 log_10_ CFU/mL difference between viable bacterial cells enumerated on drug-free and polymyxin-containing plates (8 mg/L), compared to the ~7 log_10_ CFU/mL difference seen in the controls at the same time point. Bacterial cells isolated from the control arms showed little change in polymyxin resistance profiles over the course of the IVM.

**Figure 3:**
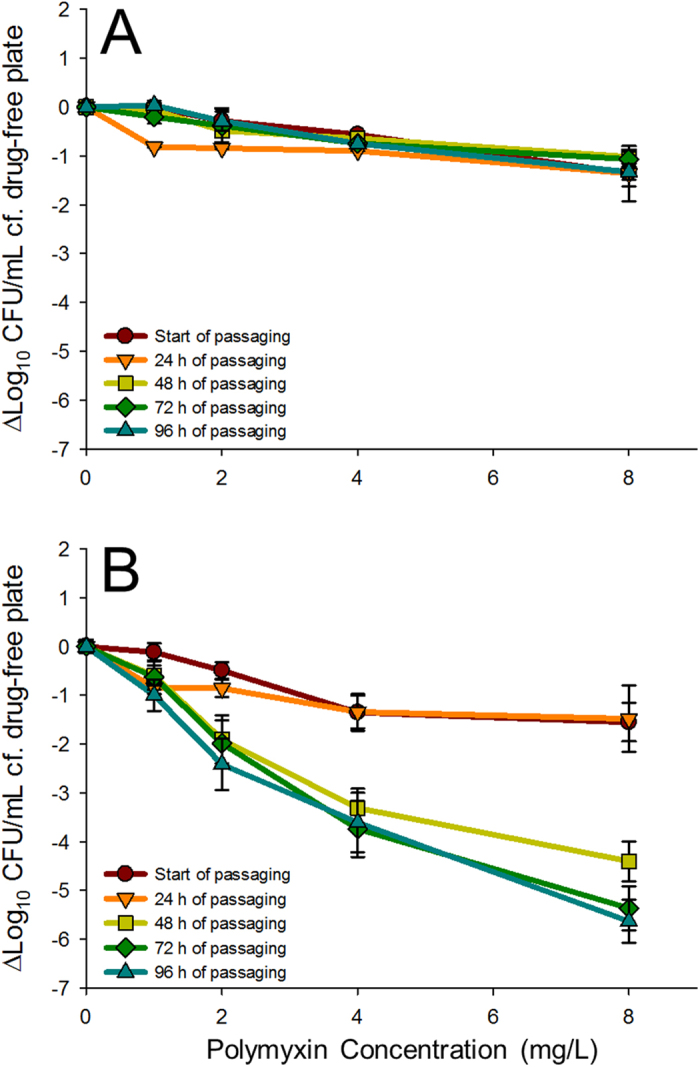
Population analysis profiles (mean ± SD; (**A**) *n* = 2; (**B**) *n* = 6) of polymyxin-treated bacterial samples during passaging in drug-free CAMHB, showing stable polymyxin resistance (**A**) and the partial reversion (>2 Log_10_ CFU/mL) to polymyxin susceptibility (**B**). Y-axis values reflect the difference in viable counts obtained on drug-free agar plates and polymyxin B containing agar plates at the concentrations indicated.

Drug-free passaging of the bacterial cells isolated from the polymyxin-treated reservoirs revealed the presence of stable (n = 2; one each from regimens 2 and 3) and non-stable (n = 6) polymyxin resistance (Fig. 3). There was insufficient evidence to strongly link the development of stable polymyxin resistance with total polymyxin exposure or dosing intensity. Further, the proportion of resistant bacteria in stable resistant bacterial samples was unchanged between the commencement and conclusion of passaging (96 h) (Fig. 3A). In the case of non-stable polymyxin resistance, partial reversion to susceptibility was extensive (>2 log_10_ CFU/mL) but incomplete over 96 h of passaging (Fig. 3B), with a ~2–3 log_10_ CFU/mL increase in viable counts on polymyxin-containing (8 mg/L) plates compared to the control.

### Genomic analysis of the stable and non-stable polymyxin resistance phenotypes

Interrogation of the genomes of the two stable polymyxin-resistant bacterial samples revealed a SNP in *pmrB* that led to a substitution of alanine at position 227 to valine (A227V). The same SNP was also found in the corresponding transcriptomic samples collected at the conclusion of polymyxin B or colistin treatment in the IVM, with >96% of reads covering the affected base containing the SNP. In all but one of the bacterial samples with non-stable polymyxin resistance, examination of the transcriptome yielded no evidence of the *pmrB* A227V mutation or any other common genomic changes across the samples. However, in the case of one non-stable polymyxin-resistant bacterial sample treated with regimen 2, 90% of transcriptomic reads covering the affected base pairs contained the *pmrB* A227V SNP.

### Transcriptomic analysis

**Figure 4:**
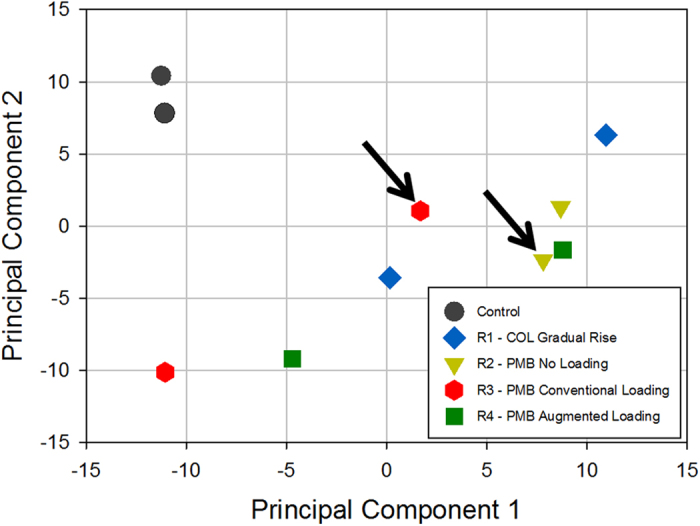
Principal components plots constructed from the transcriptomes of bacterial samples collected at the conclusion of the IVM. Arrows point to the samples exhibiting stable polymyxin resistance.

In bacterial samples collected after polymyxin B or colistin treatment for 96 h in the IVM (polymyxin-resistant cultures), 33 genes showed increased expression and 28 showed decreased expression relative to the untreated control across the four different regimens (Tables 1 and 2). Substantially increased expression (>2-fold) of *AdeA* and *AdeB* (ABBFA_001707 and ABBFA_001708, respectively), members of the *AdeABC* multidrug efflux system, was observed in all polymyxin-treated samples. Similarly, increased expression of the genes encoding components of the Lol lipoprotein transport complex (ABBFA_000739 and ABBFA_000869) and the TolQRA transmembrane complex (ABBFA_000889, ABBFA_000888 and ABBFA_000382) was also evident. GO term enrichment analysis of genes exhibiting over-expression showed a statistically significant (FDR < 0.05) over-representation of GO terms GO:0016020 (Cellular component: Membrane), GO:0005215 (Molecular function: Transporter activity), and GO:0006810 (Biological process: Transport). For the genes showing reduced expression in the presence of polymyxins, a common pattern of gene functions was less apparent, and was further confounded by the proportion of genes (12 out of 28 genes) identified as being hypothetical proteins. However, reduced expression was observed for six transcriptional regulators that have yet to be fully characterised in *A. baumannii*. No GO terms were found to be over-represented in the down-regulated gene set. Principal components analysis of transcriptomic profiles obtained at 96 h from the IVM revealed a high degree of separation between the control and polymyxin-treated (polymyxin B and colistin) samples (Fig. 4). However, a clear relationship between the dosage regimen used and the transcriptomic profile observed was not evident.

### SPLS-DA of multiple gene expression data sets

The transcriptomic data from published experiments in *A. baumannii* ATCC 19606^31^ were successfully merged and analysed with data from the current study. The validated model contained two components that included 10 and 50 genes, respectively. The early-stage transcriptomic response to polymyxin exposure was described by the first component of the SPLS model (Table 3), while commonalities between early- and late-stage responses to polymyxin exposure were characterised by the second model component (Table 4). Important predictors of early-stage polymyxin exposure in the SPLS model included genes involved in cellular metabolism (ABBFA_002620: Polyphosphate kinase, ABBFA_003493: NADPH-dependent FMN reductase family protein, ABBFA_002755: NAD^+^ synthetase) and protein mis-folding (ABBFA_002915: Peptidase C13 family protein). The genes common to early- and late-stage polymyxin exposure included seven efflux transporters (Resistance-Nodulation-Division [RND] family efflux transporters and multidrug resistance proteins A, B, and Y). Although these data are consistent with outer membrane perturbation, GO term enrichment analysis pointed to a statistically significant over-representation of only GO term GO:0009306 (Biological process: Protein secretion).

## Discussion

Given the worsening antimicrobial resistance crisis, there is an urgent need to further our understanding of the emergence of polymyxin resistance in *A. baumannii* and the association with the polymyxin exposure profile. *A. baumannii* AB307-0294, a multidrug-resistant clinical isolate from a bloodstream infection, has been genomically characterised^27^ and represents an ideal model organism for mechanistic studies into polymyxin activity and resistance in *A. baumannii*. The present study indicates that the rapid and extensive bacterial killing associated with polymyxins was dependent on rapidly attaining therapeutic concentrations (Fig. 2). Extensive polymyxin resistance was a common finding across all dosage regimens and reversion to susceptibility was substantial but incomplete during drug-free passaging in non-stable polymyxin-resistant bacterial samples (Fig. 3). Notably, there was insufficient evidence in the transcriptomic profiles to identify a clear link between the dosage regimen employed and the transcriptomic responses associated with polymyxin resistance. Complex multi-level regulatory networks are likely involved in the development of polymyxin resistance and limit the utility of transcriptomics in isolation (*i.e.* in the absence of metabolomic and proteomic studies) to characterise the mechanisms that give rise to non-stable polymyxin resistance. Further, the clinical implications of these findings will require additional investigations using *in vivo* infection models and clinical studies that adequately account for the role of immune response in infection^39^,^40^. The *in vitro* data in the present study highlight the possible limitations of polymyxin monotherapy and the need for other strategies (e.g. combination therapy) for preventing the widespread emergence of polymyxin resistance^41^.

In the present study, stable polymyxin resistance was caused by a previously documented *pmrB* A227V mutation^42^, which is hypothesized to constitutively up-regulate *pmrCAB* operon expression^17^,^21^. The notable loss of polymyxin resistance during drug-free passaging in a sample found to contain the same *pmrB* A227V mutation implicates a fitness cost associated with the mutation. This is supported by previously published studies in *pmrB* A227V mutants of *A. baumannii* strain ATCC 19606, which discovered that the mutant displayed a slower growth rate compared to wild-type strains^42^. However, the development of non-stable resistance highlights that genetic mutations are unlikely to be the sole driver of polymyxin resistance. Our findings that the transcriptomic profiles of bacteria exhibiting stable and non-stable polymyxin resistance were highly similar indicate that the stable and non-stable resistance may share a common mechanism involving *pmrB*-mediated modification of lipid A. This finding is striking in light of the conspicuous absence of the PhoPQ – PmrD signal transduction pathway in *A. baumannii* strains AB307-0294 and ATCC 19606. In other Gram-negative organisms, the PhoPQ two-component system is known to sense the presence of polymyxins and interfaces with PmrB *via* PmrD^43^. To date, an orthologous polymyxin sensing mechanism in *A. baumannii* has not been identified; there remains a pressing need to understand the role of polymyxin sensing and non-stable polymyxin resistance in determining the pharmacodynamics of polymyxin treatment in critically-ill patients.

While this study was limited to the examination of the transcriptome of *A. baumannii* strain AB307-0294, transcriptomic data from this study was successfully combined with previously published data characterising the early-stage responses to polymyxin exposure in *A. baumannii* ATCC 19606^31^. The combination of orthologous protein matching with SPLS-DA analysis enabled the integrated analysis of gene expression data between closely-related bacterial strains. SPLS-DA is a methodology developed to improve the analysis of high-dimensional omics datasets, combining multivariate statistics, dimension-reduction and feature selection^36^. This novel framework maximises the information gained from transcriptomics experiments by incorporating prior transcriptomic data. In this study, the fitted SPLS-DA model implicated the involvement of polyphosphate kinase (PPK; ABBFA002620) in the bacterial response to polymyxin exposure, a finding supported by studies in *Salmonella* that reported increased polymyxin susceptibility in Δ*PPK* mutants^44^. From these results, it can be hypothesised that the accumulation of inorganic polyphosphates is critical to the initial response to polymyxin exposure. Collectively, the altered expression of genes involved in cellular metabolism (ABBFA_003493: NADPH-dependent FMN reductase family protein, ABBFA_002755: NAD(+) synthetase) and PPK suggests that in the early stages of polymyxin exposure, the intracellular redox reactions are either directly disrupted by polymyxins or essential to the initial stress response following the exposure. Evidence of these disruptions has also been found in the metabolomes of colistin-treated *A. baumannii*^45^. Confirmation of the importance of these metabolic pathways on polymyxin activity and resistance using molecular techniques across a broader collection of *A. baumannii* strains will be crucial for identifying potential targets for novel antimicrobial agents.

GO term enrichment analysis, SPLS-DA, and a conventional analysis of gene expression showed remodelling of the OM to be a key aspect of polymyxin resistance; transcriptomic profiles obtained from *A. baumannii* strains AB307-0294 and ATCC19606 contained evidence of compensatory adaptations associated with OM remodelling. Increased expression of (RND) efflux transporter proteins (AdeABC and HlyD family) was a common finding across all analysis methodologies and stages of polymyxin exposure. This up-regulation of efflux transporters, observed in concert with over-expression of protein complexes involved in membrane homeostasis, supports previously published findings^31^ that point to the diminished integrity and barrier function of the remodelled OM in polymyxin-treated *A. baumannii*. Polymyxins have been shown to exhibit synergistic activity in combination with other antibiotics such as carbapenems and chloramphenicol^46^,^47^,^48^, and understanding polymyxin-induced OM remodelling will facilitate the development of rational antibiotic combination regimens that maximise bacterial killing and minimise the emergence of resistance.

## Conclusions

To our knowledge, this is the first study to investigate both the genomic and transcriptomic profiles of polymyxin resistance in *A. baumannii* following exposure to clinically relevant dosage regimens over an extended period. Unlike previous investigations into polymyxin resistance in Gram-negative organisms which focused on bacterial isolates that exhibit stable resistance, the present study reveals that both stable and non-stable polymyxin-resistant phenotypes are selected during treatment. Further, a framework for the integrative analysis of prior transcriptomic data from closely related bacterial strains revealed new insights into responses to polymyxins in *A. baumannii*. It remains to be elucidated the extent to which non-stable polymyxin resistance affects clinical outcomes. Our findings provide a foundation for understanding the mechanistic drivers of polymyxin resistance during polymyxin exposure resulting from clinically relevant dosage regimens, and highlight the importance of exploring optimised combination therapy in addressing the antimicrobial resistance crisis.

## Additional Information

**How to cite this article**: Cheah, S.-E. *et al.* Polymyxin Resistance in *Acinetobacter baumannii*: Genetic Mutations and Transcriptomic Changes in Response to Clinically Relevant Dosage Regimens. *Sci. Rep.* **6**, 26233; doi: 10.1038/srep26233 (2016).

## Figures and Tables

**Table 1:** Genes up-regulated in *A. baumannii* AB307-0294 following polymyxin treatment for 96 h in the IVM.

Locus Tag	Published Annotation	Annotation by Similarity Searching (Hypothetical Proteins)	Dosage Regimen (log_2_ Fold-Change)				
			Colistin (R1)	PMB No Loading Dose (R2)	PMB with Loading Dose (R3)	PMB with Augmented Loading Dose (R4)	FDR
ABBFA_000201	Succinate semialdehyde dehydrogenase	–	1.64	1.20	0.73	1.37	0.050
ABBFA_000261	Hypothetical protein	Signal peptide	3.48	2.05	2.11	3.62	0.036
ABBFA_000382	Protein TolA	–	3.43	1.21	1.92	2.71	0.044
ABBFA_000413	Hypothetical protein	Toluene tolerance protein Ttg2E	2.15	0.79	1.11	1.56	0.049
ABBFA_000570	TonB dependent receptor family protein	–	1.69	1.16	0.64	0.88	0.044
ABBFA_000739	Outer membrane lipocarrier protein LolA	–	3.43	1.18	2.01	2.74	0.044
ABBFA_000816	Multidrug resistance protein mexB	–	1.64	0.48	0.76	1.18	0.044
ABBFA_000869	Lipoprotein-releasing system transmembrane protein lolE	–	1.78	0.62	0.80	1.46	0.037
ABBFA_000870	Lipoprotein releasing system, ATP-binding protein	–	1.83	0.82	0.80	1.41	0.036
ABBFA_000885	Peptidoglycan-associated lipoprotein	–	1.25	1.00	0.65	1.08	0.050
ABBFA_000888	Protein TolR	-	1.29	0.52	0.63	1.08	0.044
ABBFA_000889	Protein TolQ	–	1.12	0.21	0.41	0.91	0.050
ABBFA_000904	Hypothetical protein	No matches	1.76	1.06	0.82	1.42	0.044
ABBFA_001022	Hypothetical protein	Putative signal peptide	2.07	0.97	1.17	1.72	0.050
ABBFA_001302	Biofilm PGA synthesis protein pgaA precursor	–	2.58	0.93	1.65	2.11	0.044
ABBFA_001303	Biofilm PGA synthesis lipoprotein pgaB precursor	–	2.43	1.01	1.40	1.95	0.044
ABBFA_001304	IcaA	–	2.25	1.11	1.30	1.85	0.044
ABBFA_001629	HTH-type transcriptional repressor Bm3R1	–	1.30	0.58	0.38	1.22	0.044
ABBFA_001662	HlyD family secretion family protein	–	1.23	1.19	0.58	0.93	0.050
ABBFA_001663	Multidrug resistance protein Y	–	1.84	1.84	1.17	1.52	0.037
ABBFA_001707	Acriflavine resistance protein E precursor	–	3.26	3.42	2.07	3.26	0.028
ABBFA_001708	AcrB protein	–	2.59	2.59	1.36	2.45	0.036
ABBFA_001709	Outer membrane protein oprM precursor	–	1.51	1.63	0.62	1.29	0.050
ABBFA_001779	UTRA domain protein	–	0.01	1.33	1.09	0.41	0.050
ABBFA_002407	Hypothetical protein	Putative signal peptide	1.62	1.54	1.00	1.62	0.036
ABBFA_002498	Putative phospholipid-binding domain protein	–	1.46	0.57	0.79	1.36	0.050
ABBFA_002880	Hypothetical protein	No matches	0.75	1.64	1.15	0.61	0.050
ABBFA_003020	Outer membrane protein oprM precursor	–	2.51	1.46	1.11	1.78	0.036
ABBFA_003050	phosphogluconate dehydratase	–	1.45	0.88	0.61	1.29	0.050
ABBFA_003147	50S ribosomal protein L31 type B	–	2.07	1.71	1.20	1.39	0.037
ABBFA_003406	AMP-binding enzyme family protein	–	1.59	0.89	0.87	1.88	0.050
ABBFA_003500	Hypothetical protein	Lipoprotein	3.28	0.64	2.07	2.69	0.050
ABBFA_003501	Hypothetical protein	Lipoprotein	2.93	1.35	1.94	3.22	0.037

**Table 2:** Genes down-regulated in *A. baumannii* AB307-0294 following polymyxin treatment for 96 h in the IVM.

Locus Tag	Published Annotation	Annotation by Similarity Searching (Hypothetical Proteins)	Dosage Regimen (log_2_ Fold-Change)				
			Colistin (R1)	PMB No Loading Dose (R2)	PMB with Loading Dose (R3)	PMB with Augmented Loading Dose (R4)	FDR
ABBFA_000133	Hypothetical protein	Ribosomal subunit interface protein	−1.05	−1.84	−1.38	−1.87	0.044
ABBFA_000218	Hypothetical protein	No matches	−1.47	−1.99	−1.48	−1.28	0.036
ABBFA_000426	Rrf2 family protein (transcriptional regulator) family protein	–	−1.70	−2.32	−1.45	−1.81	0.045
ABBFA_000555	Virulence sensor protein bvgS precursor	–	−1.60	−1.51	−1.36	−1.80	0.037
ABBFA_000602	Hemolysin-3	–	−1.56	−2.07	−1.15	−1.73	0.038
ABBFA_000699	PaaX-like family protein	–	−1.14	−1.82	−1.06	−1.28	0.044
ABBFA_000748	Hypothetical protein	No matches	−1.04	−1.29	−1.19	−1.56	0.044
ABBFA_000934	Hypothetical protein	No matches	−1.14	−1.91	−1.17	−1.02	0.038
ABBFA_000972	HTH-type transcriptional regulator gltR	–	−2.04	−2.22	−1.36	−1.89	0.050
ABBFA_000981	CRISPR-associated protein Cas1	–	−1.90	−2.65	−1.07	−1.27	0.047
ABBFA_000982	CRISPR-associated helicase Cas3	–	−1.53	−2.07	−1.12	−1.28	0.036
ABBFA_000983	Hypothetical protein	CRISPR-associated protein, Csy1 family	−1.12	−1.52	−0.96	−1.09	0.050
ABBFA_001178	Hypothetical protein	Sulphur transfer protein SirA	−1.32	−1.32	−0.67	−1.51	0.044
ABBFA_001409	Benzoate membrane transport protein	–	−0.94	−1.77	−1.12	−1.44	0.050
ABBFA_001469	Nitrogen regulation protein ntrB	–	−0.80	−1.29	−0.76	−1.24	0.038
ABBFA_001575	Hypothetical protein	Transporter component	−1.58	−1.32	−1.47	−1.55	0.039
ABBFA_001576	Hypothetical protein	Transporter component	−1.53	−1.44	−1.52	−1.69	0.047
ABBFA_001979	Hypothetical protein	No matches	−0.42	−0.99	−0.80	−1.30	0.045
ABBFA_002011	Tautomerase enzyme family protein	–	−0.81	−1.70	−0.92	−1.21	0.037
ABBFA_002314	Hypothetical protein	Lipoprotein	−1.08	−1.23	−1.01	−1.11	0.050
ABBFA_002503	Arginine exporter protein argO	–	−0.73	−1.54	−0.71	−1.01	0.044
ABBFA_002926	Hypothetical protein	Protein FilA	−0.88	−1.14	−0.86	−1.14	0.050
ABBFA_003141	Oxygen-independent coproporphyrinogen III oxidase-like protein yggW	–	−0.89	−1.02	−0.90	−1.16	0.044
ABBFA_003359	Hypothetical protein	TetR/AcrR family transcriptional regulator	−1.90	−2.19	−1.35	−1.77	0.044
ABBFA_003360	Transcription regulatory protein opdE	–	−2.62	−2.41	−1.73	−2.40	0.050
ABBFA_003361	AraC family transcriptional regulator	–	−2.35	−2.42	−1.50	−2.18	0.036
ABBFA_003470	Linoleoyl-CoA desaturase (Delta(6)-desaturase)	–	−3.91	−3.67	−2.08	−3.84	0.037
ABBFA_003471	Flavohemo (Hemoglobin-like protein)	–	−4.32	−3.92	−2.30	−3.98	0.044

**Table 3:** Genes and their inclusion frequencies (10 most frequently included shown; 300 trials) in component 1 of the SPLS-DA model and corresponding expression levels.

*A. baumannii* locus tags		Frequency of inclusion in SPLS-DA model	Annotated Product	log2-Fold Change *vs* Control		
AB307-0294	ATCC 19606			Early-Stage	Late-Stage	FDR
ABBFA_002620	HMPREF0010_01526	63.7%	Polyphosphate kinase	−1.10	0.80	1.79E-09
ABBFA_003493	HMPREF0010_03371	57.3%	NADPH-dependent FMN reductase family protein	1.67	−0.40	1.90E-10
ABBFA_000791	HMPREF0010_02052	56.0%	FtsJ-like methyltransferase family protein	0.68	−0.33	1.68E-08
ABBFA_003446	HMPREF0010_03271	50.0%	Phosphomannomutase (PMM)	1.65	−0.31	3.70E-11
ABBFA_002750	HMPREF0010_01358	42.7%	Hypothetical protein	1.13	−0.59	4.00E-10
ABBFA_002755	HMPREF0010_01353	42.7%	Probable glutamine-dependent NAD(+) synthetase	1.00	−0.13	9.09E-10
ABBFA_002915	HMPREF0010_01204	33.7%	Peptidase C13 family protein	0.74	−0.28	1.79E-08
ABBFA_003004	HMPREF0010_01698	32.3%	Hypothetical protein	1.12	−0.12	1.14E-09
ABBFA_002591	HMPREF0010_01555	32.3%	Protein hupE precursor	−0.52	0.83	1.16E-08
ABBFA_000299	HMPREF0010_02497	30.7%	Cobalt-zinc-cadmium resistance protein czcD	0.62	−0.44	1.97E-07

Component 1 of the SPLS-DA model described gene expression profiles unique to early-stage polymyxin exposure.

**Table 4:** Genes and their inclusion frequencies (Genes included in >50% of models shown; 300 trials) in component 2 of the SPLS-DA model (over 300 trials) and corresponding expression levels.

*A. baumannii* Locus Tags		Frequency of Inclusion in SPLS-DA Model		log2-Fold Change *vs* Control		
AB307-0294	ATCC 19606		Annotated Product	Early-Stage	Late-Stage	FDR
ABBFA_003136	HMPREF0010_01842	78.7%	Long-chain-acyl-CoA synthetase	1.13	1.51	1.68E-08
ABBFA_001663	HMPREF0010_00531	77.3%	Multidrug resistance protein Y	1.30	1.73	4.11E-08
ABBFA_002953	HMPREF0010_01645	77.3%	Acetyl-CoA carboxylase, carboxyl transferase, alpha subunit	−1.61	−1.73	9.23E-09
ABBFA_000610	HMPREF0010_02771	76.7%	AraC family transcriptional regulator	−1.10	−1.42	2.34E-08
ABBFA_000999	HMPREF0010_02246	76.7%	Linoleoyl-CoA desaturase (Delta(6)-desaturase)	−1.77	−1.93	1.71E-07
ABBFA_001662	HMPREF0010_00530	75.7%	HlyD family secretion family protein	0.72	1.27	2.10E-06
ABBFA_000369	HMPREF0010_02566	74.7%	CobQ/CobB/MinD/ParA nucleotide binding domain protein	−0.75	−1.00	2.38E-06
ABBFA_002706	HMPREF0010_01403	74.7%	Multidrug resistance protein B	1.51	1.89	1.16E-06
ABBFA_002838	HMPREF0010_01276	73.3%	HlyD family secretion family protein	1.36	1.58	1.83E-06
ABBFA_003296	HMPREF0010_01991	71.7%	Response regulator	−0.84	−1.17	2.81E-07
ABBFA_003340	HMPREF0010_02034	71.3%	Hypothetical protein	1.57	2.20	7.38E-05
ABBFA_001460	HMPREF0010_00336	70.3%	Hypothetical protein	−1.20	−1.42	1.41E-06
ABBFA_001969	HMPREF0010_00851	69.0%	Biotin synthase	−1.11	−1.75	2.84E-08
ABBFA_002943	HMPREF0010_01635	68.3%	Transcriptional regulator OhrR	−0.85	−1.10	5.16E-06
ABBFA_000374	HMPREF0010_02571	68.0%	Hypothetical protein	−1.50	−2.12	2.84E-07
ABBFA_002707	HMPREF0010_01402	68.0%	Multidrug resistance protein A	1.55	1.97	5.16E-06
ABBFA_001415	HMPREF0010_00288	67.3%	3-ketoacyl-(acyl-carrier-protein) reductase	1.91	1.77	1.94E-07
ABBFA_002751	HMPREF0010_01357	66.3%	Beta-ketoacyl synthase, N-terminal domain protein	−1.17	−1.74	1.16E-07
ABBFA_003341	HMPREF0010_02035	63.3%	Flavoprotein wrbA (Trp repressor-binding protein)	1.78	1.76	5.88E-05
ABBFA_000033	HMPREF0010_03386	62.7%	Efflux transporter, RND family, MFP subunit	1.49	1.57	3.91E-06
ABBFA_000205	HMPREF0010_02441	62.3%	Hypothetical protein	−1.30	−2.46	6.42E-09
ABBFA_002898	HMPREF0010_01218	61.7%	Sorbitol dehydrogenase	1.24	1.60	2.86E-05
ABBFA_002836	HMPREF0010_01278	60.3%	TetR family regulatory protein	2.28	1.71	5.54E-08
ABBFA_001549	HMPREF0010_00427	57.7%	2,4-dienoyl-CoA reductase [NADPH](2,4-dienoyl coenzymeA reductase)	1.03	2.25	7.05E-06
ABBFA_002613	HMPREF0010_01533	54.3%	Hypothetical protein	−0.87	−1.66	7.02E-07
ABBFA_000601	HMPREF0010_02763	52.0%	MFS transporter, metabolite:H+ symporter (MHS) family protein	−0.59	−0.69	5.28E-05
ABBFA_000867	HMPREF0010_02122	52.0%	Hypothetical protein	−0.43	−0.71	3.25E-05
ABBFA_000206	HMPREF0010_02442	51.7%	Methyltransferase domain protein	−0.83	−1.45	6.02E-06
ABBFA_003085	HMPREF0010_01789	51.7%	Coniferyl aldehyde dehydrogenase (CALDH)	−1.23	−1.60	3.22E-06
ABBFA_000998	HMPREF0010_02245	51.3%	Phthalate dioxygenase reductase (PDR)	−2.06	−1.88	2.64E-06

Component 2 of the SPLS-DA model described gene expression profiles common to early- and late-stage polymyxin exposure.
